# Deeply embedded esophageal fishbone removed by endoscopic submucosal dissection

**DOI:** 10.1055/a-2436-1298

**Published:** 2024-11-08

**Authors:** Sechiv Jugnundan, Sarang Gupta, Sunil Gupta, Katarzyna M. Pawlak, Christopher Teshima

**Affiliations:** 1 7938 Gastroenterology and Hepatology, University of Toronto, Toronto, Canada; 2 10071 Advanced Therapeutic Endoscopy, St Michaelʼs Hospital, Toronto, Canada

A 50-year-old man presented to the emergency department with chest pain and odynophagia after eating fish. Physical examination and X-ray of the neck were unremarkable. Computed tomography (CT) of the chest showed a fishbone embedded within the muscularis propria of the esophagus ( Fig. 1 **a** ). We then performed an endoscopic ultrasonography (EUS), which localized the fishbone to the posterior wall ( Fig. 2 ). A small mucosal defect was also noted.

**Fig. 1:**
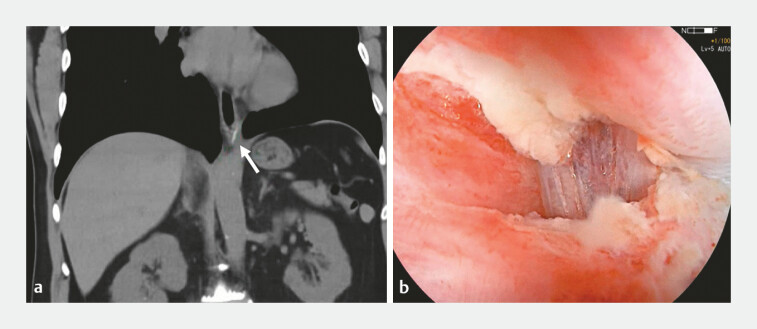
**a** Coronal slice of computed tomography showed a fishbone (arrow) lodged in the distal esophagus, that appeared to penetrate through the submucosa and muscularis propria. **b** Endoscopically a small portion of fishbone can seen embedded in the distal esophagus through the small mucosal defect.

**Fig. 2:**
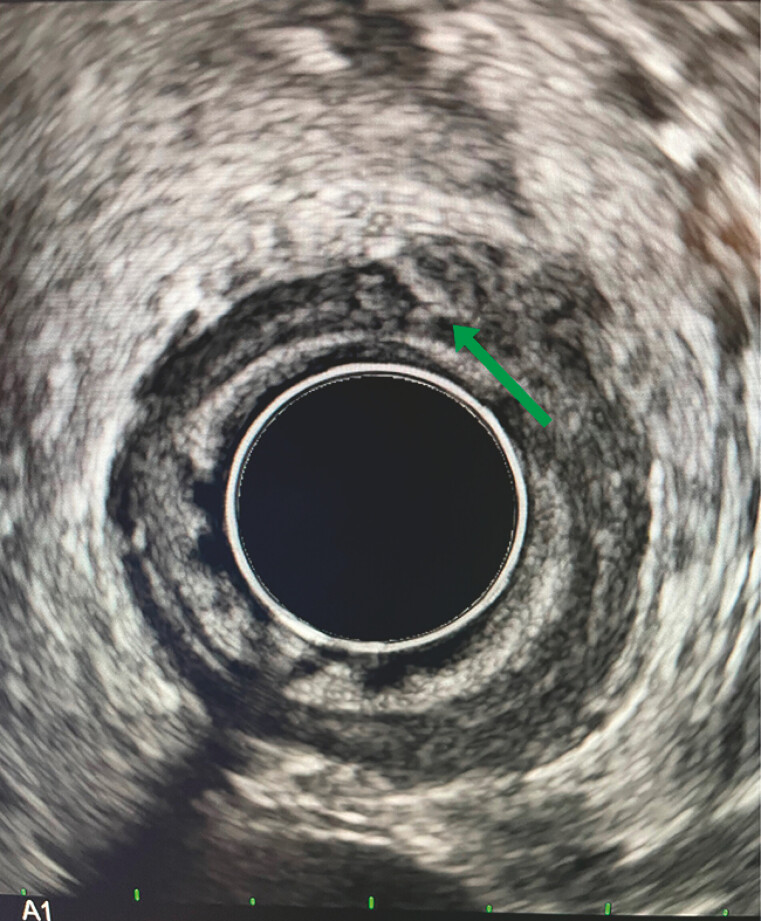
Endoscopic ultrasound showed a linear hyperdensity projecting through the muscularis propria.

We proceeded with endoscopic submucosal dissection (ESD) to remove the fishbone ( Video 1 ). After normal saline and methylene blue had been injected to expand the submucosal space, a Dual-J knife (Olympus, Center Valley, PA) was used to create a mucosal incision, laterally extending the mucosal defect ( Fig. 1 **b** ). The fishbone was exposed; it was positioned perpendicularly to the esophagus, between the circular and longitudinal muscle layers. Using the fishbone as a scaffold, the overlying circular muscle layer was progressively dissected until the right lateral tip of the bone was freed ( Fig. 3 ). The exposed tip was grasped with forceps and removed through the overtube ( Fig. 4 ). Examination of the defect revealed incised circular muscle fibers, with intact longitudinal fibers ( Fig. 5 ). Attempted closure with through-the-scope clips was unsuccessful because of the orientation of the defect. We placed a 10.5 cm × 23 mm fully covered metal stent (Wallflex; Boston Scientific, Marlborough, Massachusetts, USA). Subsequent CT 48 hours later showed appropriate positioning of the stent with no complications. The stent was removed 2 weeks later, and healed mucosa was observed.

Endoscopic submucosal dissection performed to remove a fishbone deeply embedded into the wall of the distal esophagus.Video 1

**Fig. 3:**
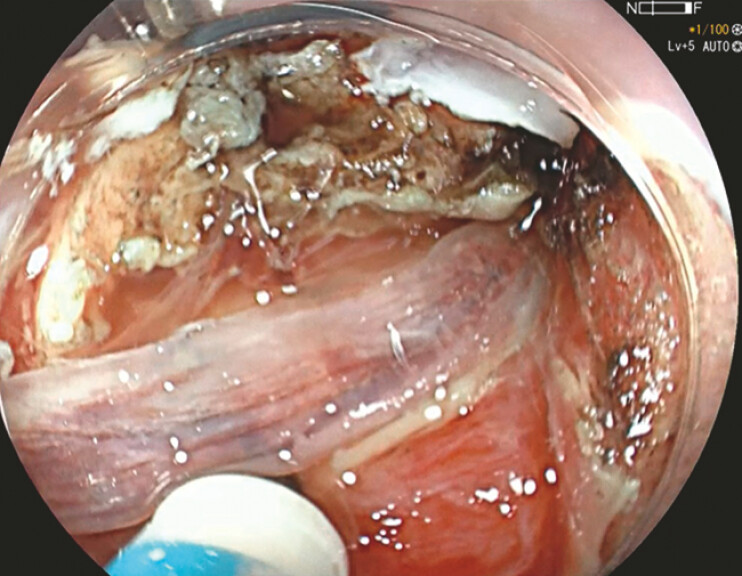
Endoscopic submucosal dissection was done along the fishbone to free the right lateral edge from the circular muscle layer.

**Fig. 4:**
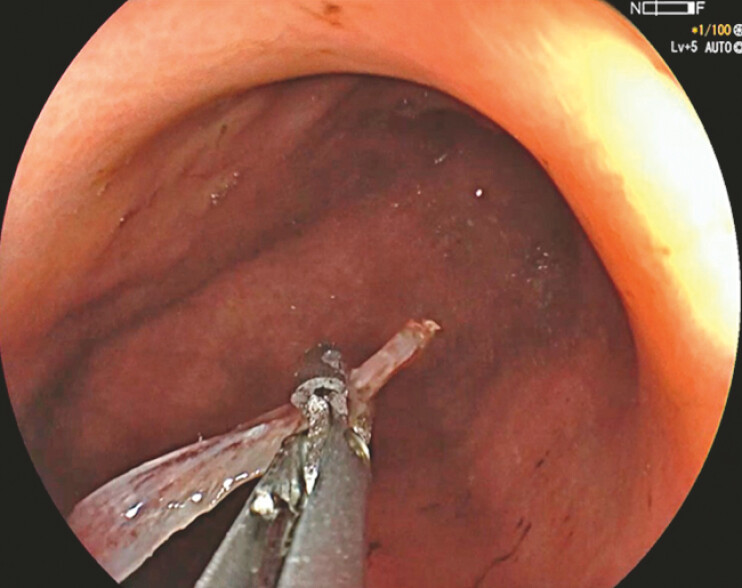
The fishbone was grasped with a rat-toothed forceps and removed via the overtube.

**Fig. 5:**
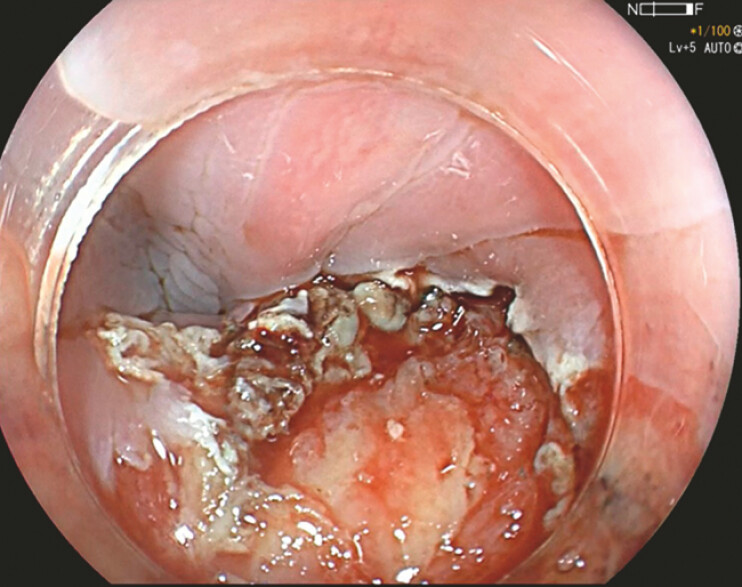
The remaining muscle defect and exposed submucosa and muscle fibers.

Foreign body ingestion is frequently encountered 1 . In adults, bony fragments are most common, and tend to become lodged at sharp angulations in the gastrointestinal tract 2 . While guidelines recommend emergent endoscopy in esophageal impaction 3 4 , surgical intervention is typically required for deeply embedded nonvisible foreign bodies 5 . As we have demonstrated, in selected patients, EUS may localize the foreign body and facilitate ESD for safe and effective extraction.

Endoscopy_UCTN_Code_TTT_1AO_2AL

## Notes

Endoscopy E-Videos https://eref.thieme.de/e-videos
*E-Videos* is an open access online section of the journal *Endoscopy* , reporting on interesting cases and new techniques in gastroenterological endoscopy. All papers include a high-quality video and are published with a Creative Commons CC-BY license. Endoscopy E-Videos qualify for HINARI discounts and waivers and eligibility is automatically checked during the submission process. We grant 100% waivers to articles whose corresponding authors are based in Group A countries and 50% waivers to those who are based in Group B countries as classified by Research4Life (see: https://www.research4life.org/access/eligibility/ ). This section has its own submission website at https://mc.manuscriptcentral.com/e-videos .
